# Gene Expression Pattern after Insertion of Dexamethasone-Eluting Electrode into the Guinea Pig Cochlea

**DOI:** 10.1371/journal.pone.0110238

**Published:** 2014-10-20

**Authors:** Yutaka Takumi, Shin-ya Nishio, Kenneth Mugridge, Tomohiro Oguchi, Shigenari Hashimoto, Nobuyoshi Suzuki, Satoshi Iwasaki, Claude Jolly, Shin-ichi Usami

**Affiliations:** 1 Department of Otorhinolaryngology, Shinshu University School of Medicine, Matsumoto, Japan; 2 Department of Hearing Implant Sciences, Shinshu University School of Medicine, Matsumoto, Japan; 3 MED-EL Headquarters, Innsbruck, Austria; University of California, Irvine, United States of America

## Abstract

A cochlear implant is an indispensable apparatus for a profound hearing loss patient. But insertion of the electrode entails a great deal of stress to the cochlea, and may cause irreversible damage to hair cells and related nerve structure. Although damage prevention effects of dexamethasone have been reported, long-term administration is difficult. In this study, we used a dexamethasone-eluting electrode in the guinea pig cochlea, and compared the gene expression after 7 days insertion with that of a normal electrode and non-surgically treated control by microarray. 40 genes were up-regulated 2-fold or more in the normal electrode group compared to the non-surgically treated group. Most of the up-regulated genes were associated with immune response and inflammation. In the dexamethasone-eluting group, compared to the normal electrode group, 7 of the 40 genes were further up-regulated, while 12 of them were down-regulated and there was a tendency to return to the non-surgical condition. 9 genes were down-regulated 2-fold or less with normal electrode insertion, and 4 of the 9 tended to return to the non-surgical condition in the dexamethasone-eluting group. These genes are certainly involved in the maintenance of the physiological functions of the cochlea. Our results indicate that the dexamethasone-eluting electrode will have an effect on the normalization of homeostasis in the cochlea.

## Introduction

Cochlear implants are widely used for patients with profound hearing loss that is not effectively remedied by use of hearing aids. Insertion of the electrode into the cochlea will potentially result in intra-cochlear injury, generally called electrode insertion trauma (EIT), and may cause irreversible loss of residual hearing due to damage to hair cells, cochlear nerve endings and spiral ganglion cells.

Notwithstanding the developments in the atraumatic surgery area, the reduction of EIT damage using synthetic glucocorticoid agonists such as dexamethasone are currently considered to be the most effective approach. Albeit generally successful, the systemic use of glucocorticoids requires both high dosage and frequent administration in order to obtain suitable therapeutic concentrations in the inner ear, therefore presenting potentially serious adverse effects to the patient characteristic of this drug class [1], [2].

To avoid such potential complications, local administration of glucocorticoids to the cochlea is now considered to be a preferable route [3] although the effectiveness of single-dose applications of these agents concurrent with cochlear implantation appears limited [4].

Although a number of studies have investigated the regulatory effects of dexamethasone on gene expression in cochlear tissue ex vivo [5]–[7] and in vivo [8] none have examined the consequence of a prolonged exposure of these tissues to this glucocorticoid. For this reason, in the current study we have utilized a dexamethasone-eluting electrode to effect a gradual (7 day) infusion of the agent into the guinea pig cochlea in order to investigate its regulatory effects on gene expression. We found that the dexamethasone-eluting electrode insertion into the cochlea reduced gene expression changes seen with implantation of the normal electrode, perhaps reflecting protective effects of dexamethasone from electrode insertion trauma.

## Materials and Methods

### Electrodes

**Figure 1:**
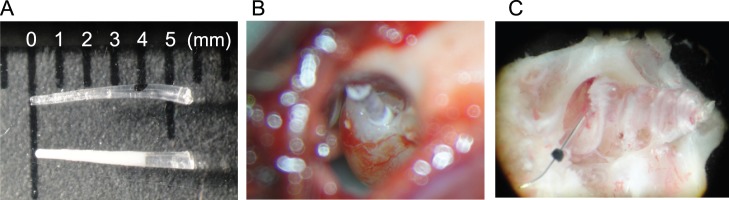
A: Microscopic view of the two types of electrode. (1) normal electrode (2) dexamethasone-eluting electrode. B: Microscopic view of the electrode insertion C: The explanted cochlea with the inserted electrode.

Both dexamethasone-eluting and normal silicone dummy electrodes were provided by Dr. Claude Jolly (MED-EL, Innsbruck, Austria). Each dummy electrode was approximately 0.5 mm in thickness and 6 mm long (Figure 1A: upper). The dexamethasone-eluting dummy displays a saturated 4 mm length of dexamethasone characterized by a clearly visible white zone (Figure 1A: lower). Pure micronized dexamethasone base was purchased from Sanofi, France. The dexamethasone was thoroughly mixed with a homogenizer with two parts silicone at 2.0% weight for weight of the final cured polymer. The prepared uncured silicone elastomer with the dexamethasone were injected in a mold designed to produce guinea pig electrode sizes and cured in an oven for 4 hours. After demolding the guinea pig electrodes with no wires and no contacts were double packaged. The packages were sterilized using ethylene oxide as with normal human electrode production and sent to Japan from Austria. The 2 part silicone was the same as that used for human cochlear implant electrodes. Consecutive batches were prepared for reproducibility analysis. Final guinea pig electrodes eluting segment were 4 mm long with elution time >1 year.

### Animals and electrode insertion

Male Hartley guinea pigs (SLC, Shizuoka, Japan) with an age of seven weeks and weighing approximately 450 g were used for the study. Following deep anaesthesia of animals by the intraperitoneal injection of 0.2 ml pentobarbitone sodium (64.8 mg/ml, Kyoritsu Seiyaku Co., Tokyo, Japan), a small hole on the left-side cochlea (near the round window) was prepared to enable insertion of the dummy electrode. Three animals (animals 1–3) were implanted with normal electrodes (3–4 mm insertion depth) while three others (animals 4–6) received a dexamethasone-eluting electrode (Figure 1B). The cochleae from two animals (animals 7 and 8), which did not undergo surgery, were used as control tissue. After completion of surgery the wound was closed by cyanoacrylate glue (Aron Alpha, Toagosei, Tokyo, Japan) and a few drops of enrofloxacin antibiotic (Baytril, Bayer Yakuhin Ltd., Tokyo, Japan) were applied to prevent infection of the wound.

### RNA extraction and probe labelling

Seven days after electrode implantation the whole temporal bone was removed and placed into RNAlater solution (Ambion, Life Technologies Co., Grand Island, NY) to stabilize and protect cellular RNA. The whole cochlea was dissected out under a microscope (Figure 1C) after which total RNA was extracted from the cochlea using an RNeasy Mini Kit (Qiagen, Hilden, Germany). The quality and quantity of the purified RNA was analysed using a BioAnalyzer 2100 (Agilent Technologies, Santa Clara, CA).

The RNA integrity number [9] for each sample was above 9.0, as determined by BioAnalyzer 2100 analysis, indicating high integrity of the RNA with little degradation (data not shown).

### CDNA synthesis and labeling

Total RNA (10 µg each) was reverse transcribed with oligo-dT primer (100 pmol) and the SuperScript Double-Stranded cDNA Synthesis Kit (Invitrogen, Life Technologies Co., Grand Island, NY) for 1 hour at 42°C. After the reverse transcription process, a second strand of cDNA was synthesized from the single strand cDNA by using he SuperScript Double-Stranded cDNA Synthesis Kit according to the manufacturer’s procedure (Invitrogen) and after second strand synthesis, total RNA was removed by using RNaseA. Labeled cDNA was synthesized by using a NimbleGen One-Color DNA Labeling Kit (Roche NimbleGen Inc., Madison, WI) including cyanine 3-CTP labeled primer and Klenow enzyme (3′->5′ exo-) according to the manufacturer’s instructions. Labeled cDNA was purified by ethanol precipitation and quantified by the NanoDrop Spectrophotometer (Thermo Scientific Wilmington, DE).

### Hybridization to the microarray, data Scanning and analysis

To analyze the gene expression, the 3 normal electrode samples, 3 dexamethasone-eluting electrode samples, and 2 untreated samples were hybridized to the Eukaryote gene expression array for *Cavia porcellus* (Roche NimbleGen) respectively. This oligonucleotide microarray covers guinea pig 13,684 transcripts (13684 genes). Four µg of Cy3-labeled cDNA was hybridized to a microarray slide and washed according to the manufacturer’s procedure.

Fluorescence intensities were measured with the Agilent Microarray Scanner (Agilent Technologies) using the scanning protocols specific for each microarray assay and raw microarray image files were created. The expression data were extracted from raw microarray image files using Agilent Feature Extraction Image Analysis Software (Version 10.7.3.1, Agilent Technologies). The signal intensities for a single gene measured by different probes, including alternative splicing variants, were analyzed as one gene. The average of gene expression signals for the non-surgically treated control and normal electrodes were compared using Student’s *t*-test. The microarray data was available in the Gene Expression Omnibus (http://www.ncbi.nlm.nih.gov/geo/) as accession number: GSE53866.

### Quantitative RT-PCR

To confirm the microarray analysis results, quantitative real-time RT-PCR was performed on 9 genes. Reverse transcription was performed with 10 ng of total RNA from the 3 normal electrode samples, 3 dexamethasone-eluting electrode samples, and 2 untreated samples, respectively, using a High Capacity RNA-to-cDNA Kit (Applied Biosystems, Life Technologies, Foster City, CA) according to the manufacturer’s protocol. Quantitative RT-PCR primers for the amplification of the DNA fragment including the splicing junction region were designed using the Primer3Plus website (http://www.bioinformatics.nl/cgi-bin/primer3plus/primer3plus.cgi/). Primers used in the quantitative RT-PCR are shown below:

GAPDH (NM_001172951): CAGTGACAGCCATTCTTCCA/TGGGGTCCACTTACTCCTTG.
ACTB (NM_001172909): CTGTGGCATCCACGAAACTA/GCTGGAAGGTGGAGAGTGAG.
IL15 (NM_001172829): TTACATGAGTCCAGAAATGAAGACA/GCAAAAATTCTGCAATGCTT.
LPL (NM_001172978): CTACACGGAAGTGGACATCG/GCAGCTTCGACACTTTCTCC.
TRPC6 (NM_001173032): AGCGATGAGGTGAATGAAG/TTGCTTTGGTTCTGAGGACA.
LTB4R (NM_001172835): AAAGTGTGCATCGCTTTGG/ATGCCCTTCACAGTCTGAGC.
C4BPA (NM_001173110): CACTGTTGTCGTCTGAAGCA/CTCGGAGAACCCTCCAATTT.
TGFB1 (NM_001173023): TGGAGCCTGGACACACAGTA/ACGATCATGTTGGAGAGCTG.
IL1B (NM_001172968): GATAACAAAATGCCCGTTGC/GCGGATTCAAATTCCACTGT.

Quantitative RT-PCR was performed 3 times for each sample with Fast SYBR Green Master Mix (Applied Biosystems, Life Technologies) and a StepOne Plus real-time PCR system (Applied Biosystems, Life Technologies) according to the manufacturers’ instructions. In brief, 10 µl of Fast SYBR Green Master Mix, 0.5 µl of the 10 pM PCR primers, 0.5 µl of the cDNA samples and distilled water were added to 96-well plates to give a final reaction volume of 20 µl and then amplified for 20 sec at 95°C, followed by 40 two-step cycles of 95°C for 3 sec and 60°C for 30 sec. After every PCR cycle, the fluorescence intensity of SYBR green dye was measured using the StepOne Plus real-time PCR system. GAPDH was chosen as an internal control gene. The estimated gene expression level (EL) was normalized to the GAPDH expression level, and data are presented as the mean of log2EL.

### Ethics Statement

All experimental procedures were performed in accordance with the regulations for animal experimentation of Shinshu University. These experiments were approved by Shinshu university institutional animal care and use committee.

## Results

### Scatter plot analysis of gene expression profiles in guinea pig cochlea

**Figure 2:**
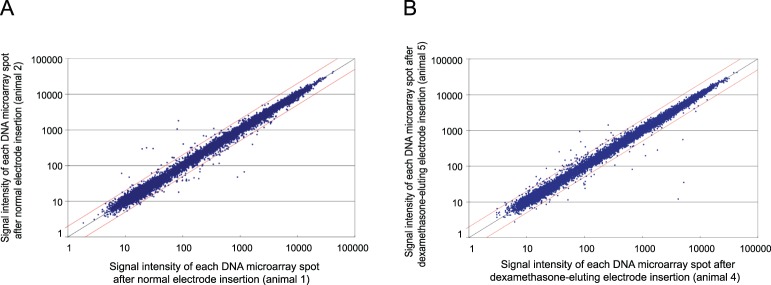
Scatter plot analysis of gene expression profiles in guinea pig cochlea. Both panels show scatter plots using identical type of electrode in different animals to verify the technical stability. A: normal electrode (animal 1) compared to normal electrode (animal 2) B: dexamethasone-eluting electrode (animal 4) compared to dexamethasone-eluting electrode (animal 5).

Figure 2 illustrates the uniformity of scatter plots obtained from identical types of electrodes in two each of the three animals from the normal group (animals 1 and 2) and the dexamethasone-eluting group (animals 4 and 5). Most of the gene expression was quite similar between the different animal samples, revealing that the surgical and RNA extraction procedure in this study were quit stable and had minimal effect on later analysis.

**Figure 3:**
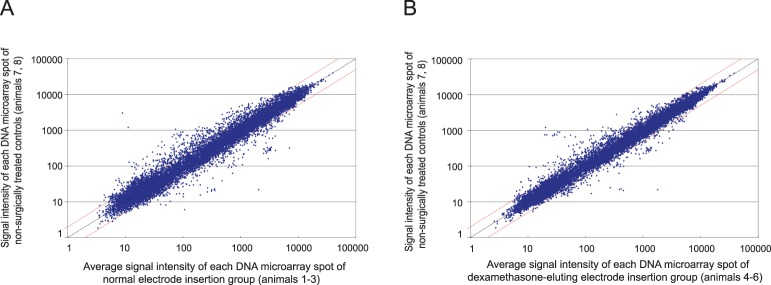
Scatter plot analysis of gene expression profiles of normal electrode (NOR) compared to without electrode (CNT) (A) and dexamethasone-eluting electrode (DEX) compared to without electrode (CNT) (B). The suppression of various gene expressions by dexamethasone-eluting electrode was demonstrated (B).

Figure 3 indicates the suppression effect by dexamethasone elution on the gene expression change seen in normal electrode insertion. The left-hand panel (A) demonstrates the scatter plot arising from the implantation of a normal electrode compared to a non-surgically treated control. In contrast, the right panel (B) demonstrates the scatter plot obtained from the implantation of a dexamethasone-eluting electrode compared to the same non-surgically treated control. An overall suppression of gene expression due to dexamethasone is clearly present as seen by the general tightening of the scatter plot.

### Genes that are up- or down-regulated by normal electrode insertion into the cochlea

At first, we compared the gene expression profiles between the normal electrode group and the non-surgically treated control group to elucidate the effect of the cochlea implantation surgery on the guinea pig cochlea gene expression. 40 genes were up-regulated 2-fold or more in the cochlea by normal electrode insertion (Table 1), and most of the up-regulated genes were associated with immune response and inflammation (including interleukin-1 beta, interleukin-15, eotaxin, RANTES (CCL5), gro (CXCL1), tumor necrosis factor-alpha, transformin growth factor-beta, monocyte chemoattractant protein-1, cyclooxygenase-2, prostaglandin E2 receptor, Fc-gamma receptor, leukotriene B4 receptor, interleukin-15 receptor, toll like receptor 3, MHC class II, CD-1b1, CD1-b4, CD1-e, and CD8 alpha, CD8 beta). On the other hand, 9 genes, including neutrophil cationic peptide, were down-regulated 2-fold or less in the cochlea with normal electrode insertion (Table 2).

**Table 1:** Genes up-regulated 2-fold or more by normal electrode insertion compared to non-surgically treated controls (40 genes).

		AveragedSignalintensity			NOR vs CNT		DEX vs CNT		DEX vs NOR	
Symbol	Genefunction	Ave. NOR	Ave. DEX	Ave. CNT	FoldChange	*P* value	FoldChange	*P* value	FoldChange	*P* value
LPL	lipoprotein lipase	2631.6	1411.4	146.0	18.03	0.00	9.67	0.03	0.54	0.02
C4BPA	C4bp alpha-chain	205.9	283.7	16.3	12.66	0.00	17.44	0.00	1.38	0.24
FABP5	E-FABP	2687.5	2270.9	263.1	10.22	0.00	8.63	0.07	0.84	0.52
TRPC6	trpc6	137.2	56.1	17.4	7.87	0.00	3.22	0.00	0.41	0.02
CCL2	monocyte chemoattractantprotein-1	1291.3	1803.1	196.4	6.58	0.04	9.18	0.10	1.40	0.43
GPIFCGRB1	Fc-gamma-1/gamma-2 receptor precursor	2244.8	3463.8	441.5	5.08	0.03	7.84	0.05	1.54	0.21
GPIFCGRB2	Fc-gamma-1/gamma-2 receptor precursor	2586.2	4023.7	586.5	4.41	0.04	6.86	0.03	1.56	0.14
GPIFCGRB3	Fc-gamma-1/gamma-2 receptor precursor	113.1	200.0	26.5	4.27	0.05	7.56	0.08	1.77	0.19
IL-1β	interleukin-1 beta	407.5	233.6	97.0	4.20	0.05	2.41	0.11	0.57	0.21
F3	tissue factor	776.8	463.8	219.8	3.53	0.01	2.11	0.12	0.60	0.04
TNF-α	tumor necrosis factor alpha	52.3	40.1	17.4	3.01	0.01	2.30	0.08	0.77	0.19
GPR34	G protein-coupled receptor 34	537.6	532.6	181.2	2.97	0.01	2.94	0.05	0.99	0.85
IL-15	interleukin-15 precursor	80.0	74.9	27.0	2.96	0.05	2.77	0.09	0.94	0.82
TGM	transglutaminase	1933.4	1316.6	665.0	2.91	0.04	1.98	0.15	0.68	0.17
LTB4R	leukotriene B4 receptor	131.4	75.1	45.6	2.88	0.05	1.65	0.05	0.57	0.17
CD8-α	CD8 alpha	162.2	142.0	56.8	2.86	0.00	2.50	0.05	0.88	0.40
TBXAS1	thromboxane synthase	76.7	77.0	27.1	2.84	0.00	2.85	0.01	1.00	0.97
MHC class II	major histocompatibilitycomplex class II	3954.3	2860.4	1412.1	2.80	0.00	2.03	0.02	0.72	0.02
CXCL1	GRO	297.4	482.4	109.0	2.73	0.00	4.43	0.11	1.62	0.22
HIF1-α	HIF1-alpha protein	476.4	323.9	177.1	2.69	0.01	1.83	0.05	0.68	0.05
NCX1	sodium/calcium exchangerisoform NaCa3	58.3	53.8	23.0	2.53	0.01	2.34	0.00	0.92	0.47
IL15Ra	interleukin-15 receptoralpha chain precursor	193.0	238.4	79.2	2.44	0.02	3.01	0.09	1.23	0.44
COX-2	cyclooxygenase-2	16.4	14.2	6.8	2.42	0.01	2.09	0.23	0.87	0.61
11βHSD	11-beta-hydroxysteroiddehydrogenase type 1	97.5	115.1	40.3	2.42	0.02	2.85	0.04	1.18	0.28
TGF-β	transforming growthfactor-beta	691.1	511.6	294.1	2.35	0.01	1.74	0.08	0.74	0.06
C3AR1	anaphylatoxin C3a receptor	1979.9	1853.9	855.6	2.31	0.00	2.17	0.00	0.94	0.65
CD1-e	CD1-E	37.1	47.7	16.1	2.30	0.01	2.96	0.44	1.29	0.72
CCL5	RANTES	123.6	101.4	53.9	2.29	0.02	1.88	0.01	0.82	0.38
CD1-b1	CD1-B1	18.9	18.6	8.3	2.27	0.05	2.23	0.17	0.98	0.95
FBN1	fibrillin 1	2450.3	1966.7	1082.3	2.26	0.00	1.82	0.22	0.80	0.34
PTGER4	prostaglandin E2 receptor 4	21.0	23.5	9.4	2.24	0.00	2.50	0.18	1.12	0.71
CD18	leukocyte adhesion glycoproteinMo1-alpha	223.3	262.2	102.0	2.19	0.02	2.57	0.12	1.17	0.56
AO	leukotriene b4 12-hydroxydehydrogenase/prostaglandin15-keto reductase	672.0	603.2	310.4	2.17	0.04	1.94	0.15	0.90	0.64
CYBA	flavocytochrome b-558alpha polypeptide	3707.4	3079.6	1727.7	2.15	0.05	1.78	0.00	0.83	0.25
L-asparaginase	L-asparaginase	5318.1	6220.7	2485.3	2.14	0.01	2.50	0.00	1.17	0.14
CD1-b4	CD1-B4	13.1	14.2	6.1	2.14	0.01	2.31	0.22	1.08	0.80
CD-8β	CD8 beta	102.0	88.4	47.9	2.13	0.00	1.84	0.04	0.87	0.23
C4BPA	C4bp alpha-chain	11.2	11.6	5.4	2.09	0.04	2.17	0.01	1.04	0.79
TLR3	toll-like receptor 3	21.7	17.3	10.6	2.06	0.01	1.64	0.15	0.80	0.23
FMO5	flavin-containing monooxygenase 5	482.1	430.0	235.0	2.05	0.05	1.83	0.19	0.89	0.65

Abbreviations: Ave. NOR, average of expression signals from animals that received normal electrode (No. 1–3); Ave. DEX, average of expression signals from animals that received dexamethasone-eluting electrode (No. 4–6); Ave. CNT, average of expression signals from animals with no treatment (No. 7, 8); NOR vs CNT, fold change of normal electrode/non-surgically treated control; DEX vs CNT, fold change of dexamethasone electrode/non-surgically treated control; DEX vs NOR, fold change of dexamethasone electrode/normal electrode.

**Table 2:** Genes down-regulated 2-fold or less by normal electrode insertion compared to non-surgically treated controls (9 genes).

		Signal intensity			NOR vs CNT		DEX vs CNT		DEX vs NOR	
GENE_NAME	FUNCTION	Ave. NOR	Ave. DEX	Ave. CNT	FoldChange	*P* value	FoldChange	*P* value	FoldChange	*P* value
ngb	neuroglobin	47.76	57.43	95.12	0.50	0.00	0.60	0.01	1.20	0.13
KCNH2	potassium channelprotein	264.03	256.15	577.56	0.46	0.02	0.44	0.02	0.97	0.81
sca1	spinocerebellar ataxiatype 1 protein	61.82	79.17	136.35	0.45	0.01	0.58	0.08	1.28	0.29
PrRPR	prolactin releasingpeptide receptor-likeprotein	19.56	25.43	43.93	0.45	0.00	0.58	0.07	1.30	0.35
GPM4	muscarinic receptor 4	13.18	17.16	30.67	0.43	0.05	0.56	0.03	1.30	0.28
sperad-7	sperad-7	111.60	141.82	318.32	0.35	0.02	0.45	0.04	1.27	0.23
PRM1	protamine 1	27.96	31.74	85.49	0.33	0.02	0.37	0.02	1.14	0.43
sperad-4	sperad-4	352.40	404.50	1441.20	0.24	0.03	0.28	0.04	1.15	0.64
TAC2	beta preprotachykinin I	22.50	23.28	997.46	0.02	0.01	0.02	0.01	1.03	0.92

Abbreviations: see Table 1.

### Genes that are up- or down-regulated by dexamethasone elution

**Figure 4:**
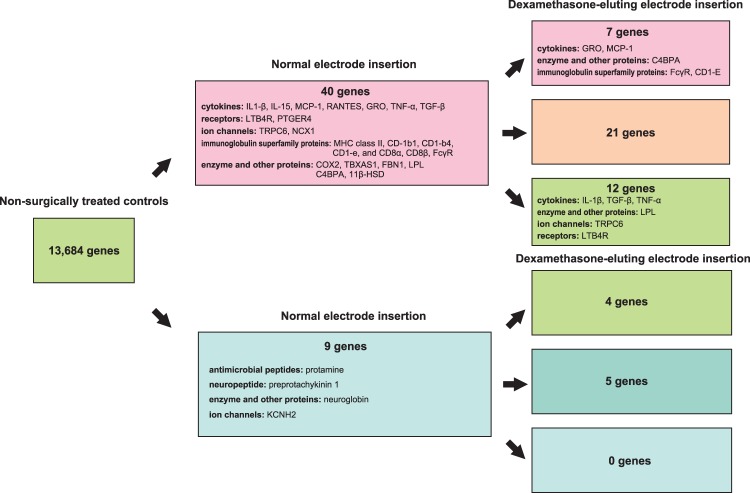
Changes in up- and down-regulation of gene expression with insertion of normal and dexamethasone-eluting electrode insertion. Up-regulation was 2-fold or more, and down-regulation was 2-fold or less, with normal electrode insertion compared to non-surgically treated controls. Up-regulation was 1.25-fold or more, and down-regulation was 1.25-fold or less, with dexamethasone-eluting electrode insertion compared to normal electrode insertion.

To elucidate the effect of the electrode that elutes dexamethasone in order to protect the cochlea after insertion, gene expression profiles were compared between the normal electrode insertion and dexamethasone-eluting electrode insertion groups (Figure 4). Of the 40 genes that were up-regulated in the normal electrode group, in the dexamethasone-eluting group, 7 of them (including Fc-gamma receptor, gro, and c4bp) were further up-regulated 1.25-fold or more, while 12 of them (including leukotriene B4 receptor, interleukin-1 beta, and trpc6) were down regulated 1.25-fold or less, partially returning them to the non-surgical condition (Table 1). On the other hand, of the 9 genes that were down-regulated in the normal electrode group, 4 were up-regulated 1.25-fold or more in the dexamethasone group partially returning them to the non-surgical condition, but none were down-regulated 1.25-fold or less (Table 2). These results were consistent with the scatter plot analysis results, clearly presenting the suppression effect by dexamethasone elution on the gene expression change that was seen in normal electrode insertion. Quantitative RT-PCR was performed for 7 genes. The expression patterns of those genes obtained by microarray analysis closely coincided with those obtained by the quantitative RT-PCR (Table 3).

**Table 3:** Changes in gene expression confirmed by quantitative RT-PCR.

	Fold change in geneexpression bymicroarray analysis		Fold change in geneexpression byquantitative RT-PCR	
	NOR/CNT	DEX/NOR	NOR/CNT	DEX/NOR
LPL	18.03	0.54	22.34	0.49
C4BPA	12.66	1.38	10.51	1.58
TRPC6	7.87	0.41	18.07	0.45
IL1B	4.20	0.57	6.41	0.42
IL15	2.96	0.94	3.40	0.87
LTB4R	2.88	0.57	4.76	0.84
TGFB	2.35	0.74	2.23	0.78

## Discussion

In the present study, a novel dexamethasone-eluting electrode [10] has been used to examine the influence of a sustained release of this agent on gene expression patterns in the guinea pig cochlea.

### Up- or down-regulated gene expression by normal electrode insertion

The 40 genes listed in Table 1 were up-regulated 2-fold or more in the cochlea by normal electrode insertion. Included in those up-regulated genes were many kinds of cytokine which are well known to associate with immune response, such as interleukin-1 beta (IL1-β), interleukin-15 (IL15), monocyte chemoattractant protein-1 (CCL2), RANTES (CCL5), gro (CXCL1), tumor necrosis factor-alpha (TNF-α), and transformin growth factor-beta (TGF-β). Many receptors and enzymes associated with inflammation response, for instance, cyclooxygenase-2 (COX2), thromboxane synthase, prostaglandin E2 receptor, and leukotriene B4 receptor, were also up-regulated 2-fold or more. And many kinds of immunoglobulin superfamily proteins expressed in lymph cells associated with antigen presentation and immune globulin production or killer T cell activation were also up-regulated (MHC class II, CD-1b1, CD1-b4, CD1-e, CD8α, CD8β and Fc γ receptors). These results clearly indicated that that normal electrode insertion associated trauma caused the immune response including lymphocytic infiltration and inflammation reaction including activation of arachidonic acid cascade. On the other hand, 9 genes, including antimicrobial peptides (protamine 1) were down-regulated 2-fold or less in the cochlea with normal electrode insertion.

### Dexamethasone suppression effect of electrode insertion trauma

Among the 40 genes that were up-regulated in the normal electrode insertion, 12 were down-regulated 1.25-fold or less in the dexamethasone-eluting electrode insertion, partially or completely returning them to the non-surgical condition (Table 1, Figure 4). Among the 9 genes that were down-regulated 2-fold or less in the cochlea with normal electrode insertion, 4 were up-regulated 1.25-fold or more in the dexamethasone-eluting electrode insertion, also partially returning them to the non-surgical condition (Table 2, Figure 4).

The relevance of a selected number of the gene expression changes is discussed in the following sections. In general, dexamethasone-eluting electrode insertion into the cochlea reduced gene expression changes seen with implantation of the normal electrode, perhaps reflecting normalizing effects of dexamethasone on electrode insertion trauma.

#### Receptors

Leukotriene B4 (LTB4) is a powerful chemoattractant and activator of both neutrophils and eosinophils [11], [12]. Neutrophils rapidly release high amounts of LTB4 in response to various activating stimuli and have a high density of cell-surface LTB4 receptors [13]. Implantation of the normal electrode induced about 3-fold increases in LTB4R expression, and demonstrated about 50% reduction of the total increase in LTB4 receptor expression with the dexamethasone-eluting electrode. This may plausibly represent a means to regulate blood flow as well as to reduce inflammatory cell recruitment into the tissue.

#### Ion channels

The transient receptor potential (trp) gene superfamily encodes cation channels that act as sensors for a wide variety of stimuli from both inside and outside the cell. They transduce electrical and calcium signals through their cation channel activities. In the cochlea, TRPC6 appears essential for normal mechanotransduction since TRPC6 knock-out mice demonstrated hearing impairment, vestibular deficits and defective auditory brain stem responses to high-frequency sounds [14]. Implantation of the normal electrode induced over 8-fold increases in TRPC6 expression, possibly as a result of the surgical trauma. Dexamethasone elution was observed to partially reverse this increase by 60%. It is likely that this is a pro-survival action by the glucocorticoid since TRPC6 has been associated with podocyte apoptosis [15], and that dexamethasone reverses this process via blocking TRPC6 channel expression [15].

#### Enzymes and proteins

An 18-fold increase in the expression of lipoprotein lipase (LPL), a key enzyme involved in lipid metabolism, was observed following insertion of the normal electrode. LPL is present throughout the central nervous system and peripheral nerves [16] although, to date, its expression has not been reported in the cochlea. Its presence may be due, at least in part, to macrophages which have migrated to the insertion site as a response to trauma, this being observed previously in a rat model of nerve crush injury [17]. LPL expression in the cochlea was partially reduced (45%) by dexamethasone elution. Increased expression of LPL in the cochlea following insertion of the normal electrode is most likely due to insertion trauma. And although dexamethasone treatment might reduce LPL expression, suppressed ingress of both monocytes and macrophages are known sources of LPL into the cochlea. Dexamethasone has been reported to reduce monocyte recruitment in the rat [18] and importantly has been demonstrated to reduce macrophage presence following electrode insertion in the cochlea of guinea pig [19].

#### Cytokines

IL-1 is comprised of two principal 17 kDa polypeptides, IL-1α and IL-1β encoded by genes found on chromosome 2 [20]. Over-expression of IL-1β is considered as a major factor leading to the general amplification of inflammatory responses and has been described in over-expression of cisplatin- [21] and salicylate-induced ototoxicity [22], and acoustic trauma [23]. Insertion of the normal electrode in the guinea pig cochlea increases IL-1β expression more than 4-fold over untreated animals. Although dexamethasone elution did not reduce IL-1 β expression to the level found in untreated animals, being still 4-fold higher, it did reduce (40%) the elevated expression caused by insertion of the normal electrode. Dexamethasone is well documented to interfere with the synthesis of IL-1β, an action thought to be at the transcriptional level of the IL-1 gene [24] involving blocking NFKB and activator protein 1 (AP-1) activation [25] with mRNA destabilization [26].

### Genes further up-regulated by dexamethasone elution

As summarized in Figure 4, 40 genes were up-regulated 2-fold or more by the normal electrode insertion surgery, and 7 of them were further up-regulated 1.25-fold or more in the dexamethasone-eluting electrode insertion. On the other hand, none of the 9 genes that were down-regulated 2-fold or less in the cochlea with normal electrode insertion were down regulated 1.25-fold or less with dexamethasone-eluting electrode insertion (Figure 4). In context, the role of these genes in the cochlea and the significance of their up-regulation by dexamethasone may give important information regarding the mechanisms by which this agent affords protection and assists in maintaining the physiological functions of the cochlea.

#### Enzymes and other proteins

C4b-binding protein (C4BP) is a large glycoprotein synthesized mainly in the liver and is thought to interact with the complement system where it acts as an inhibitor. C4BP binds apoptotic and necrotic cells [27], [28]. C4BP expression was found in the cochleae of untreated animals and was dramatically increased, more than 13-fold, as a result of normal electrode insertion and increased even further by dexamethasone elution. Dexamethasone has been demonstrated to increase the expression of C4BP, this action also being considered as a means to down-regulate complement activation and exert control over the inflammatory process [28].

#### Cytokines

Monocyte chemoattractant protein-1 (MCP-1), designated as CCL2 is implicated in the pathogeneses of several diseases that are typified by monocytic infiltration [29]. CCL2 has been considered as one of the most proven effectors of monocyte chemotaxis in vivo [30]. Implantation with the non-eluting electrode stimulated CCL2 expression almost 6.5-fold while dexamethasone treatment increased this further to over 9-fold compared to non-surgically treated controls. Dexamethasone has been observed to decrease CCL2 expression in spiral ligament fibrocytes [5] but has also been reported to be ineffective in human visceral adipose tissue [31] suggesting that its effects are cell specific. In fact, there is evidence that the CCR2 participates in protection against noise-induced cell death [32] and the expression of CCL2 is increased in this type of trauma [33]. It is feasible that increased CCL2 expression provoked by both normal and dexamethasone-eluting electrodes constitutes a protective mechanism for the hair cells.

Growth-regulated oncogene (GRO)α, GROβ, GROγ are a family of proteins involved in modulating inflammatory responses. Three distinct GRO isoforms (α, β, and γ) have been identified and now are referred to as CXCL1, CXCL2, and CXCL3 respectively. Insertion of the normal electrode into the guinea pig cochlea elevated expression, 2.7-fold, of GRO precursor with dexamethasone further increasing expression 4.3-fold. Previous studies have observed that dexamethasone both inhibits [34] and increases [35] CXCL1 expression. It is not clear why dexamethasone increases expression levels of the eotaxine and GRO precursor in the cochlea and this requires further elucidation.

#### Receptors

Fc γ receptors (FcγR) belong to the immunoglobulin superfamily and are expressed on the surface of a number of cells including macrophages, neutrophils, and mast cells. They are considered the most important family member for stimulating phagocytosis of opsonized microbes [36]. Implantation alone with the normal electrode was sufficient to promote increased expression, circa 4–5-fold higher than that of untreated animals, of the Fc-gamma-1/gamma-2 receptor precursor which was further increased, more than 7-fold, by dexamethasone elution. Such an increase may be indicative of an infection being present, possibly as a result of the surgical procedure.

## Conclusion

The present study revealed the gene expression profile of dexamethasone-eluting electrode insertion, showing the suppression of various gene expressions compared to the normal electrode. These results suggest that the dexamethasone-eluting electrode will have an effect on the normalization of homeostasis in the cochlea.
